# In Vivo Biokinetics of ^177^Lu-OPS201 in Mice and Pigs as a Model for Predicting Human Dosimetry

**DOI:** 10.1155/2019/6438196

**Published:** 2019-01-03

**Authors:** Seval Beykan, Melpomeni Fani, Svend Borup Jensen, Guillaume Nicolas, Damian Wild, Jens Kaufmann, Michael Lassmann

**Affiliations:** ^1^Department of Nuclear Medicine, University of Würzburg, Würzburg, Germany; ^2^Division of Radiopharmaceutical Chemistry, University Hospital Basel, Basel, Switzerland; ^3^Department of Nuclear Medicine, Aalborg University Hospital, Aalborg, Denmark; ^4^Department of Chemistry and Bioscience, Aalborg University, Aalborg, Denmark; ^5^Division of Nuclear Medicine, University Hospital Basel, Basel, Switzerland; ^6^Octreopharm Science GmbH, Ipsen Group, Berlin, Germany

## Abstract

**Introduction:**

^177^Lu-OPS201 is a high-affinity somatostatin receptor subtype 2 antagonist for PRRT in patients with neuroendocrine tumors. The aim is to find the optimal scaling for dosimetry and to compare the biokinetics of ^177^Lu-OPS201 in animals and humans.

**Methods:**

Data on biokinetics of ^177^Lu-OPS201 were analyzed in athymic nude *Foxn1* ^nu^ mice (28 F, weight: 26 ± 1 g), Danish Landrace pigs (3 F-1 M, weight: 28 ± 2 kg), and patients (3 F-1 M, weight: 61 ± 17 kg) with administered activities of 0.19–0.27 MBq (mice), 97–113 MBq (pigs), and 850–1086 MBq (patients). After euthanizing mice (up to 168 h), the organ-specific activity contents (including blood) were measured. Multiple planar and SPECT/CT scans were performed until 250 h (pigs) and 72 h (patients) to quantify the uptake in the kidneys and liver. Blood samples were taken up to 23 h (patients) and 300 h (pigs). In pigs and patients, kidney protection was applied. Time-dependent uptake data sets were created for each species and organ/tissue. Biexponential fits were applied to compare the biokinetics in the kidneys, liver, and blood of each species. The time-integrated activity coefficients (TIACs) were calculated by using NUKFIT. To determine the optimal scaling, several methods (relative mass scaling, time scaling, combined mass and time scaling, and allometric scaling) were compared.

**Results:**

A fast blood clearance of the compound was observed in the first phase (<56 h) for all species. In comparison with patients, pigs showed higher liver retention. Based on the direct comparison of the TIACs, an underestimation in mice (liver and kidneys) and an overestimation in pigs' kidneys compared to the patient data (kidney TIAC: mice = 1.4 h, pigs = 7.7 h, and patients = 5.8 h; liver TIAC: mice = 0.7 h, pigs = 4.1 h, and patients = 5.3 h) were observed. Most similar TIACs were obtained by applying time scaling (mice) and combined scaling (pigs) (kidney TIAC: mice = 3.9 h, pigs = 4.8 h, and patients = 5.8 h; liver TIAC: mice = 0.9 h, pigs = 4.7 h, and patients = 5.3 h).

**Conclusion:**

If the organ mass ratios between the species are high, the combined mass and time scaling method is optimal to minimize the interspecies differences. The analysis of the fit functions and the TIACs shows that pigs are better mimicking human biokinetics.

## 1. Introduction

Recently, the radiolabeled somatostatin receptor subtype 2 (SST2) agonists DOTA-[Tyr3]octreotate (DOTATATE), DOTA-[Tyr3]octreotide (DOTATOC), and DOTA-[NaI3]octreotide (DOTANOC), as well as the antagonists OPS201 (DOTA-JR11) and OPS202 (NODAGA-JR11), have been used for imaging and treatment of neuroendocrine tumors (NETs) which are overexpressing the somatostatin receptor SST2 [1–5].

Previous preclinical and clinical studies have indicated that radiolabeled SST2 antagonists are superior to the corresponding agonists especially for tumor targeting despite little to no internalization in tumor cells [1, 3, 5–7]. A possible explanation for this observation is that the antagonistic peptides are independent of the somatostatin receptor activation state (G-protein phosphorylation); therefore, they utilize more binding sites on the tumor cell surface, have a lower dissociation rate, and also have longer tumor retention than agonistic peptides [8]. It was also shown that the uptake in the tumor is higher for SST2 antagonists compared to SST2 agonists [1, 3–5, 8]. The absorbed dose to the kidneys, the main organ at risk after treatment of NETs with DOTA labeled compounds [9], was around 50% higher for the antagonist as compared to the agonist ^177^Lu-octreotate [5].

Rodents are the most frequently used species in preclinical studies. However, larger animals such as pigs or dogs are expected to mimic humans' physiology better than rodents [2]. In addition, these larger animals can be scanned several times with a human SPECT/CT under the same conditions as patients. Therefore, these studies have the advantage of long follow-up times and showed that multiple blood samples can be taken for dosimetry and metabolism assessment similar to patient studies.

Until today, there is one clinical human study (by Wild et al. [5]), two preclinical mouse model studies (by Dalm et al. [7] and by Nicolas et al. [3]), and one preclinical pig study (by Beykan et al. [2]) with ^177^Lu-DOTA-JR11 (OPS201) focusing on biodistribution and dosimetry [2, 3, 5, 7]. In all of these studies, the main focus was on biodistribution and dosimetry. In the clinical study [5], the dosimetry of four patients with advanced NET was analyzed and compared to ^177^Lu-DOTATATE. In the preclinical study by Dalm et al. [7], tumor-xenografted mice were used to determine the optimal dosage for therapy, and the therapeutic effect of ^177^Lu-OPS201 (^177^Lu-DOTA-JR11) was compared to the effect of ^177^Lu-DOTA-octreotate. The follow-up period of the experiments was short (4 time points up to 7 d after injection) for a quantification of the biodistribution and dosimetry. In another preclinical study on mice by Nicolas et al. [3], OPS201 labeled with ^177^Lu, ^90^Y, and ^111^In was compared with the ^177^Lu-DOTATATE. Neither time-integrated activity coefficient (TIAC) values nor absorbed dose values were published; only the relative administered activity values per gram were reported. The focus of the preclinical pig study by Beykan et al. [2] was on in vivo biodistribution and dosimetry in pigs. Five pigs (four with coadministered amino acids and one without kidney protection) were analyzed; TIAC, absorbed dose, and effective dose coefficients values were reported.

For dose calculations, none of the preclinical studies accommodated methods for considering the differences in physiology between animals and humans. For this purpose, extrapolation methods are used that are based on mathematical equations in order to predict TIACs and consequently absorbed doses in humans by using data collected from animals. Mostly, these techniques are needed for predicting the absorbed doses for a first application of a radiopharmaceutical in humans [10]. As of today, there is no systematic study that analyzes the difference in biokinetics of radiopharmaceuticals dedicated to therapy between animal models and patients. In total, there are five published interspecies extrapolation methods related to the use of radionuclides [10, 11] in preclinical studies. However, there are no studies related to either comparing extrapolation methods or optimizing a scaling method.

Therefore, the aim of this work is to compare the in vivo biokinetics of ^177^Lu-OPS201 in two animal models (mice and pigs) and in patients for the liver, kidneys, and blood. In addition, all published extrapolation methods related to the use of radionuclides (“scaling methods”) were examined to find the optimal method for analyzing biokinetics and dosimetry.

## 2. Methods

OPS201 was synthesized and ^177^Lu-OPS201 was prepared for mice as described in the study by Nicolas et al. (for mice [3]), by Beykan et al. (for pigs [2]), and by Wild et al. (for humans [5]).

For analyzing the biokinetics of ^177^Lu-labeled peptides in preclinical and clinical studies, the data of ^177^Lu-OPS201 athymic nude Foxn1nu mice (28 females, weight: 26 ± 1 g, age: 8–9 weeks) [3], Danish Landrace pigs (3 females-1 male, weight: 28 ± 2 kg, age: 3 months) [2], and patients (3 females-1 male, weight: 61 ± 17 kg, age: 44–77 years) [5] with administered activities of 0.19–0.27 MBq (mice, 0.017 *µ*g of peptide), 97–113 MBq (pigs, 9 *µ*g of peptide), and 850–1086 MBq (patients, 55–106 *µ*g of peptide) were included. For pigs and patients, kidney protection was applied.

After administration of ^177^Lu-OPS201, blood samples were taken up to 72 h for mice, up to 300 h for pigs, and up to 23 h for patients in order to measure the blood radioactivity contents by using the same well-type gamma counter (Packard Instruments). The human blood data were, originally, provided as relative values, normalized to the first blood sample immediately taken after injection. In order to convert the raw count values to blood uptake values per mL human blood in each time point, human blood data were quantified (in Bq/mL) retrospectively by using the same calibration factor as for the mouse study. As the data showed high variability, the median values of all mice and patients were used for further processing.

### 2.1. Image Acquisition and Reconstruction for Liver and Kidneys

#### 2.1.1. For Pigs

After injection, multiple whole body (WB) planar images and SPECT/CT scans were acquired at 0.5, 2, 3, 4, 50, 100, 150, and 250 h to quantify the uptake in the kidneys and liver. SPECT/CT data and WB planar images were acquired using Symbia T16 (Siemens AG). The acquisition duration was 50 min for all scans: 10 min for WB and 40 min for SPECT (2 bed positions of 20 min each). In addition, a 5 min CT was performed for attenuation correction. For reconstruction, CT-based attenuation correction and triple energy window-based scatter corrections were applied. The images were reconstructed with the FLASH 3D iterative reconstruction algorithm with 6 iterations and 6 subsets. The resulting images were smoothed with a 6 mm Gauss filter.

#### 2.1.2. For Patients

SPECT/CT data and WB planar images were generated with the Philips BrightView XCT equipped with a medium-energy, parallel-hole collimators SPECT/CT scanner. WB scans and low-dose SPECT/CT were performed at 1, 3, 24, and 72 h after 975 MBq mean administered activity of ^177^Lu-OPS201. The acquisition duration was 43 min for all scans: 17 min for WB and 26 min for SPECT (2 bed positions of 13 min each). In addition, a CT was performed for attenuation correction. For reconstruction, CT-based attenuation correction and triple energy window-based scatter corrections were applied. The images were reconstructed with the Astonish (Philips) iterative reconstruction algorithm with 4 iterations and 16 subsets.

### 2.2. Dosimetry Analysis

#### 2.2.1. Quantification of Activity and Integration of the Time-Activity Curves

To quantify the amount of activity, the average percentage values corresponding to the injected radioactivity (*A*%) per organ as a function of time were calculated for the liver, right kidney, left kidney, and blood for each species via a manual VOI analysis (for pigs and patients) and via gamma counter (for mice). For pigs, all VOIs were drawn based on the CT scan. In order to avoid spill-out effects, CT-based organ VOIs were enlarged as matching 2 voxels plus their actual CT-based volumes. For mice, scarified organs were counted by using the well-type gamma counter, total numbers of count values for the selected organs (kidneys and liver) were reported, and *A*% values were calculated. The time-activity curves of blood for each species were analyzed separately from the collected samples. Since the scan times of pigs were not exactly identical to those of all animals, the population-based *A*% values of pigs were used to create the time-activity curves for both selected organs and blood. For the mice and humans, since all scanning time points were identical in each study and the standard deviations in each time point are less than 5% (as is shown in the supplementary file), mean *A*% values for organs and median *A*% values for blood were used.

To analyze the interspecies differences in biokinetics of ^177^Lu-OPS201, the time-dependent uptake data sets for the kidneys, liver, and blood were used, and individual fits (TACs) for each species including optimal fit function parameters by using the software solution NUKFIT [12] were created. The resulting fits were investigated to compare the biokinetics of the different species.

The organ TIACs were calculated by integration of the mean (for mice and humans) and population-based (for pigs) time-dependent uptake data sets using NUKFIT [12], choosing the optimal fit functions as proposed by the code. The TIACs are estimated by analytically integrating the fitted functions. Their standard error values are determined assuming Gaussian error propagation (can be seen in Supplementary Table 1). For this investigation, a systematic error in activity quantification of 10% was assumed for each measured data point.

### 2.3. Extrapolation Methods

There are several extrapolation approaches that are used to estimate TIAC values, absorbed doses, and in vivo biokinetics and biodistribution in humans based on animal data. Assuming the same biodistribution in animals and humans is one of the most commonly used methods, which means applying no extrapolation. In addition to this, relative mass scaling, time scaling, allometric scaling, and the combined relative mass and time scaling are the other techniques described in the literature; however, there is no common well-accepted method.

In this study, five interspecies extrapolation methods were applied on blood TIAC values (only for pigs) and kidneys and liver TIAC values (for mice and pigs) and examined to determine the optimal method for dosimetry [10, 13]. None of the extrapolation method could be applied on blood TIAC values of mice since the data for the total animal blood volume were not available.

Method 1 (equation (1)) (“same biodistribution approach”) is based on the assumption that the TIACs for the same organ in an animal and human are the same [10]. Method 2 (equation (2)) is relative mass scaling in which the TIAC value of a human organ is set equal to the TIAC value of the same animal organ multiplied by the ratio of WB and the selected organ mass of the human and animal. Method 3 (equation (3)) is time scaling in which time is scaled by a power function of the ratio of WB masses of the human and animal for calculating the TIACs. In Method 3, the exponent is set to 0.25 [10]. Method 4 is a combined method: first time scaling is applied (equation (3)) and then the TIAC values of the animal are scaled based on relative mass scaling (equation (2)) [10]. Method 5 (equation (4)) applies allometric scaling in which TIACs of an animal are scaled by a power function of the ratio of WB masses of the human and animal. In this method, the exponent depends on the selected organ and is set equal to 0.92 for the liver and 0.82 for the kidneys [13]:

(1) $$\begin{array}{c} \text{TIAC}\;\;{\text{organ }}_{\text{human}}=\text{TIAC}\;\;{\text{organ }}_{\text{animal}}, \end{array}$$

(2) $$\begin{array}{c} \text{TIAC}\;\;{\text{organ }}_{\text{human}}=\text{TIAC}\;\;{\text{organ }}_{\text{animal}}\;\;\times \frac{{\left(m_{\text{organ}}/m_{\text{WB}}\right)}_{\text{human}}}{{\left(m_{\text{organ}}/m_{\text{WB}}\right)}_{\text{animal}}}, \end{array}$$

(3) $$\begin{array}{c} t_{{\text{organ}}_{\text{human}}}=t_{{\text{organ}}_{\text{animal}}}\;\;\times {\left[\frac{{\left(m_{\text{WB}}\right)}_{\text{human}}}{{\left(m_{\text{WB}}\right)}_{\text{animal}}}\right]}^{1/4}, \end{array}$$

(4) $$\begin{array}{c} \text{TIAC}\;\;{\text{organ }}_{\text{human}}=\text{TIAC}\;\;{\text{organ}}_{\text{animal}}\times {\left[\frac{{\left(m_{\text{WB}}\right)}_{\text{human}}}{{\left(m_{\text{WB}}\right)}_{\text{animal}}}\right]}^{\left[b-1\right]}, \end{array}$$

where *m* = mass, WB = whole body, *t* = time, *b* = scaling component, *b* (for liver) = 0.92, and *b* (for kidneys) = 0.85.

## 3. Results

### 3.1. Biodistribution and Dosimetry Calculations

**Figure 1:**
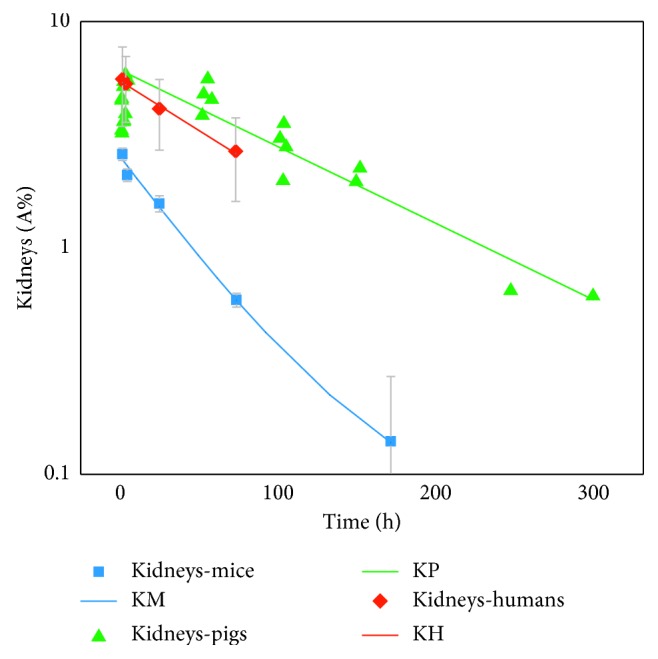
Time-activity curves of the kidneys based on VOI and well-type gamma counter analysis for each species. Dots: time-dependent percentage uptake data sets for the kidneys. Line: fit curves for the kidneys including fit function parameters from NUKFIT for mice (KM), pigs (KP), and humans (KH), respectively. All standard deviation values were less than 10% (can be seen in Supplementary Tables 2(a) and 2(b)).

**Figure 2 fig2:**
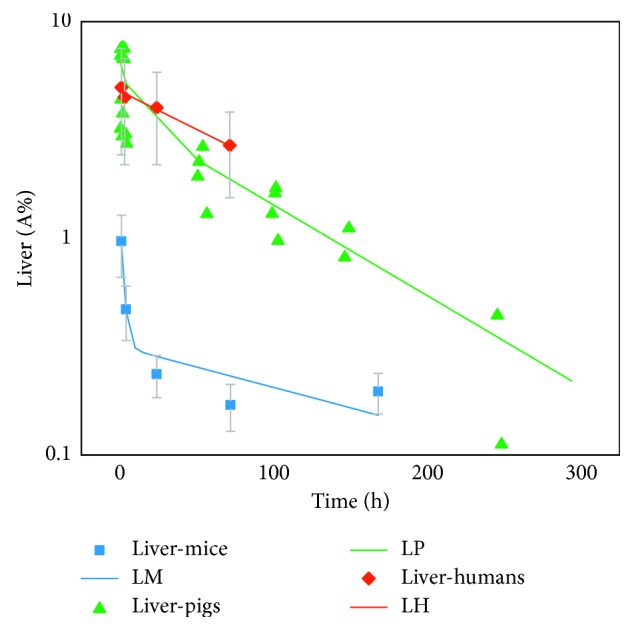
Time-activity curves of the liver based on VOI and well-type gamma counter analysis for each species. Dots: time-dependent percentage uptake data sets for the liver. Line: fit curves for the liver including fit function parameters from NUKFIT for mice (LM), pigs (LP), and humans (LH), respectively. All standard deviation values were less than 10% (can be seen in Supplementary Tables 2(a) and 2(b)).

**Figure 3:**
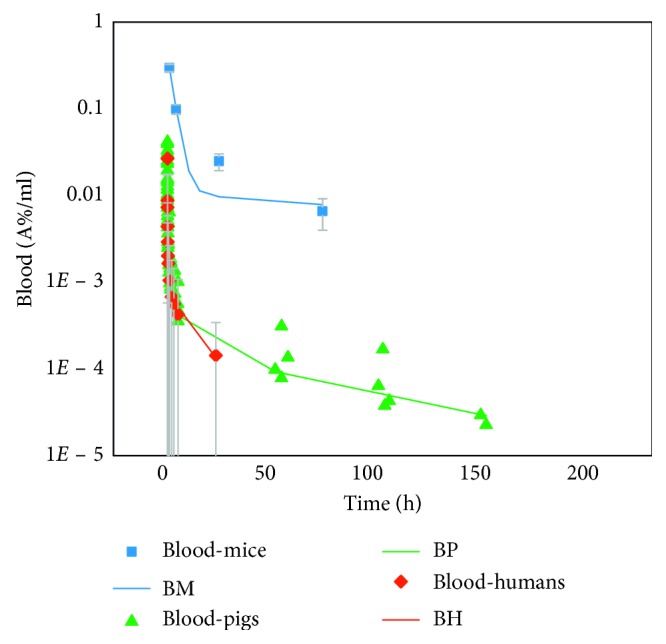
Time-activity curves of blood based on VOI and well-type gamma counter analysis for each species. Dots: time-dependent percentage uptake data sets for the blood. Line: fit curves for the blood including fit function parameters from NUKFIT for mice (BM), pigs (BP), and humans (BH), respectively. All standard deviation values were less than 10% (can be seen in Supplementary Tables 2(a) and 2(b)).

Calculated lambda values used to create the TACs by using the optimal fit function parameters from NUKFIT for each species and organ are shown in Supplementary Table 1. The respective species-dependent time-activity curves based on VOI and well-type gamma counter analysis for the kidneys, liver, and blood are displayed in Figures 1 [Fig fig2]–3. Dots represent time-dependent percentage uptake data sets for the selected organs and blood, while lines represent individual fits (TACs) including fit function parameters from NUKFIT for the selected organs and blood in each species. Kidneys, liver, or blood fit curves including fit functions were named using the first letter of the kidneys, liver, or blood such as for mice (KM, LM, or BM), pigs (KP, LP, or BP), or humans (KH, LH, or BH), respectively. Since a logarithmic scale was used in all figures for better visualization, the error bars cannot be distinguished in total. However, all standard deviation values were less than 10% (shown in Supplementary Tables 2(a) and 2(b)). A fast blood clearance of the compound is observed in the first phase (largest half-life: 1.83 h; Supplementary Table 1) for each species. 10 min after injection, less than 5% of the injected activity per milliliter of blood circulates in pigs and humans (Figure 3). Overall, the blood clearance of OPS201 in pigs and humans was faster compared to mice.

The best approximation for the last phase of the liver curves for pigs and humans was a monoexponential function. However, the liver decay function in mice has biexponential characteristics. The slope of the last phase was lower than the corresponding function of pigs and humans. In comparison to patients, pigs show higher liver retention (Figure 2). As for kidneys, the shapes of curves for each species were similar (Figure 1).

The resulting TIACs based on extrapolation methods are summarized in Tables 1 and 2. Applying Method 1 (same biodistribution approach) to the mice data for both kidneys and liver resulted in underestimation by a factor of 4 for the kidneys and a factor of 7 for the liver compared to the patient data (kidney TIAC: mice = 1.4 h and patients = 5.9 h; liver TIAC: mice = 0.7 h and patients = 5.3 h). On the contrary, since pigs mimic humans better as compared to mice, Method 1 in pigs results in a slight overestimation for the kidneys and a slight underestimation for the liver by a factor of 1.3 (kidney TIAC: pigs = 7.7 h and patients = 5.9 h; liver TIAC: pigs = 4.1 h and patients = 5.3 h).

Most similar TIACs were obtained by applying time scaling (Method 3) and combined relative mass and time scaling (Method 4) methods (kidney TIAC: mice = 3.9 h, pigs = 4.8 h, and patients = 5.9 h; liver TIAC: mice = 0.9 h, pigs = 4.7 h, and patients = 5.3 h; Table 2). Other methods showed higher deviations.

The kidney TIAC values of mice (except the results after applying Method 3) are underestimated approximately fourfold in Method 1 and Method 4, and they are overestimated twelvefold by Method 2 (relative mass scaling) and Method 5 (allometric method). In contrast to the mouse data, the kidney TIAC values of pigs did not show high levels of variations; the data are overestimated 1.2–1.6 times in Method 1, Method 3, and Method 5.

For mice liver TIAC values despite of the scaling, even when applying Method 3, underestimations approximately by a factor of 6 (in Method 3) up to 17 (in other methods) were observed. For pigs liver TIAC values, only in Method 2 and Method 4, underestimations approximately by a factor of 5 were calculated; other applied methods show similar results.

## 4. Discussion

In this study, the in vivo biokinetics of ^177^Lu-OPS201 for three species (mice, pigs, and humans) in the liver, kidneys, and blood were compared by using well-type gamma counter measurements and multiple WB planar and SPECT/CT images. In addition to this, all applicable scaling methods in the literature were summarized in order to identify an appropriate extrapolation method that minimizes the interspecies differences for comparing biokinetics, in vivo biodistribution, and dosimetry.

Observed interspecies differences in the fitted curves used to investigate the biokinetics show the necessity of scaling. Five interspecies extrapolation methods were tested on the kidneys and liver of both species (mice and pigs) and also on blood data of pigs. Our results show that all applied scaling methods, except time scaling (Method 3), result in a weight-dependent decrease of TIAC values and, consequently, the absorbed doses. Instead of Method 1, when the organ mass ratios between the species are high (e.g., for mice compared to humans), the scaling method either 3 or 4 should be applied to predict in vivo biokinetics, dosimetry, and absorbed doses in humans based on animal data more accurately. On the contrary, in small animals like mice, despite the applied extrapolation methods, interspecies differences may still be observed. For instance, in our study, none of the applied extrapolation methods on mice liver TIAC values provides similar values compared to humans due to the biphasic clearance of the OPS201 agent from the mice liver which was different compared to pigs and humans.

Although mouse models are applied widely in cancer translational research, there are still some limitations that need to be addressed [14]. Amongst others, the main differences in physiological parameters are the organ size, the heartbeat rate, and, as a consequence, the faster biological half-life of radioactive compounds in the animals [14]. In addition, gender-specific differences may play a role; however, the setup of the studies was not optimized to address these potential effects.

Allometric scaling may account partially for some of these effects as we have shown in our study (equation (4); Method 5). However, as de Jong and Maina stated [14], it is advisable to remain “critical and cautious about the applicability of animal data to the clinical domain.”

Not only scaling but also the follow-up period plays an important role when investigating the biokinetics of therapeutic agents. In this study, the follow-up time in mice and patients was rather short, especially for an analysis of the biokinetics and dosimetry. The blood samples were taken from 1 h up to 72 h for mice and from 0.3 h up to 23 h for patients. We are missing the early phase (for mice) and late phase (for mice and humans) of the biokinetics. These data at early time points provide valuable information of the uptake pattern of the radiopharmaceutical, whereas for biodistribution and dosimetry assessments of ^177^Lu-labeled compounds, the late time points (72 hours and later) have the greatest impact on the TIAC values which directly affect also the absorbed dose values [15]. In order to have sufficient data leading to more accurate results for analyzing the biokinetics and dosimetry, blood sampling at least up to 150–200 h is needed. As observed for blood, additional data on both early time points and late time points are needed for a better analysis of the liver and kidney biokinetics in mice and patients, despite the fact that the patterns in each species were similar.

Since the follow-up time of blood in mice and humans was not sufficient and, additionally, because of high variability in the median values for humans and mice blood data, mice blood data were neglected from the extrapolation method analyses. In addition, there is a lack of information about the total blood volume of mice; thus, the uptake of the radiopharmaceutical cannot be deduced. On the contrary, since we do not have these limitations in pigs, five extrapolation methods were applied on the pig blood data set. In addition to this, in pigs, measurements could be carried out over a longer period for dosimetry, biokinetics, and biodistribution assessments of therapeutic agents as compared to rodents, which makes the analyses more stable and accurate.

Since the kidneys and bone marrow are critical organs in ^177^Lu-OPS201 treatment, applying our results to calculate bone marrow-absorbed doses could potentially improve the study analyses. Bone marrow dosimetry can be performed either on the basis of blood and whole body TIACs [16, 17] image based on scans of lumbar vertebrae 2–4 (LV2–4) [18]. As we have neither data for mice on the activity contents of bone marrow containing tissues nor LV2–4-segmented uptake values for the patients, a comparison of bone marrow dosimetry based on images (for humans) and on bone marrow uptake values (for mice) could not be performed. For the blood-based method, the main contributor to the bone marrow-absorbed dose is the TIAC of the blood ([16, 17]) which we have compared in our work. For future studies, it could be beneficial to have bone marrow tissue samples and/or corresponding image data for an improved comparison of bone marrow TIACs and, as a consequence, absorbed doses.

The fast blood clearance of the OPS201 in the first phase (<56 h) for each species was in agreement with studies of the agonist [15, 19]. Sandstrom et al. [15] observed a first phase with a mean effective half-life of 1.6 h, in agreement with our data for mice (1.8 h) and pigs (1.7 h). For humans, most likely because of the short observation period, the value was lower (0.5 h). For the late phase in pigs, our result (58 h) is also close to the results obtained in the human study with the agonist (43 h). Part of an ongoing phase 1 study [20] with ^177^Lu-OPS201 in patients with SSTR-positive progressive NETs, in which dosimetry data are taken also at time points later than 48 h, is to substantiate whether the biokinetics of the agonist and antagonist in the pig model are comparable to those in the patients after treatment with ^177^Lu-OPS201.

## 5. Conclusion

Extrapolation methods need to be applied in preclinical studies in order to predict the biokinetics, TIACs, absorbed doses, and dosimetry in humans more accurately. According to our results, if the organ mass ratios between the species are high (e.g., for mice compared to humans), the most adequate scaling method for TIACs is either time scaling or combination of relative mass and time scaling. Furthermore, this study shows that, for the ^177^Lu-labeled dosimetry studies, follow-up times at late time points (more than 72 h) are needed for TIAC calculations in order to appropriately represent the area under the curve and to analyze both biokinetics and dosimetry accurately. Based on our analysis of the biokinetics, fit functions, and the TIAC values, pigs mimic humans better than mice. In addition to all of these topics mentioned above, increasing the number of subjects and including a gender-based analysis of biokinetics and dosimetry may produce even more representative results.

## Figures and Tables

**Table 1:** Time-integrated activity coefficient (TIAC (unit: h)) values for the selected organs of mice, pigs, and humans with respective error calculated by NUKFIT with an assumption of 10% systematic error based on Method 1 (same biodistribution approach).

	Kidney TIAC ± error (h)	Liver TIAC ± error (h)	Blood TIAC ± error (ml/h)
*Method 1*			
Mice	1.44 ± 8.5*E* − 02	0.75 ± 4.1*E* − 02	0.0370 ± 2.0*E* − 03
Pigs	7.67 ± 1.8*E* − 01	4.08 ± 9.4*E* − 02	0.0002 ± 3.4*E* − 06
Humans	5.85 ± 4.2*E* − 01	5.32 ± 3.4*E* − 01	0.0002 ± 8.2*E* − 05

**Table 2:** Time-integrated activity coefficient (TIAC (unit: h)) values for the selected organs of mice and pigs based on applied scaling methods.

	Kidney TIAC (h)		Liver TIAC (h)		Blood TIAC (h/ml)
	Mice	Pigs	Mice	Pigs	Pigs
Method 2	0.43	4.17	0.32	1.04	0.00022
Method 3	3.89	8.73	0.88	4.65	0.00025
Method 4	1.17	4.75	0.38	1.18	0.00026
Method 5	0.44	6.63	0.40	3.78	0.00021

Method 2: relative mass scaling; Method 3: time scaling; Method 4: combined relative mass and time scaling; Method 5: allometric scaling.

## Data Availability

The data used to support the findings of this study are available from the corresponding author upon request.

## Abbreviations

*A*%: The average percentage values corresponding to the injected radioactivity
CT: Computed tomography
JR11: Cpa-c(DCys-Aph(Hor)-DAph(Cbm)-Lys-Thr-Cys)-DTyr-NH_2_
^177^Lu: Lutetium 177
NET: Neuroendocrine tumor
OPS201: DOTA-JR11
OPS202: NODAGA-JR11
PRRT: Peptide receptor radionuclide therapy
SD: Standard deviation
SST2: Somatostatin receptor subtype 2
TIACs: Organ-specific time-integrated activity coefficients
TACs: Individual time-activity fits
VOI: Volume of interest
WB: Whole body.

## Supplementary Materials

Supplementary Materials Supplementary Table 1: calculated lambda values used to create the TACs by using the optimal fit function parameters from NUKFIT [12] for each species' organs and blood including standard deviation (SD) and coefficient of variation (CV) values determined assuming Gaussian error propagation. Supplementary Table 2(a): the median and mean percentage injected radioactivity (*A*%) values per ml in blood of mice and humans with corresponding standard deviation (SD). Supplementary Table 2(b): the mean percentage injected radioactivity (*A*%) values per ml in the kidneys and liver of mice and humans with corresponding standard deviation (SD).
