# Supplementary material for: In Vivo Biokinetics of 177Lu-OPS201 in Mice and Pigs as a Model for Predicting Human Dosimetry
Source: Contrast Media Mol Imaging. 2019 Jan 3;2019:6438196. doi: 10.1155/2019/6438196 (PMC6348830; doi:10.1155/2019/6438196)
Supplement: Supplementary Materials — Supplementary Table 1: calculated lambda values used to create the TACs by using the optimal fit function parameters from NUKFIT [12] for each species' organs and blood including standard deviation (SD) and coefficient of variation (CV) values determined assuming Gaussian error propagation. Supplementary Table 2(a): the median and mean percentage injected radioactivity (A%) values per ml in blood of mice and humans with corresponding standard deviation (SD). Supplementary Table 2(b): the mean percentage injected radioactivity (A%) values per ml in the kidneys and liver of mice and humans with corresponding standard deviation (SD). [file 6438196.f1.pdf]

In-vivo biokinetics of ^{177}Lu-OPS201 in mice and pigs as a model for predicting human dosimetry

Supplementary file

Beykan Seval[1], Fani Melpomeni[2], Jensen Svend Borup[3,4], Nicolas Guillaume[5], Wild Damian[5], Kaufmann Jens[6], Lassmann Michael[1]

1: Department of Nuclear Medicine, University of Würzburg, Würzburg, Germany

2: Division of Radiopharmaceutical Chemistry, University Hospital Basel, Basel, Switzerland

3: Department of Nuclear Medicine, Aalborg University Hospital, Aalborg, Denmark

4: Department of Chemistry and Bioscience, Aalborg University, Aalborg, Denmark

5: Division of Nuclear Medicine, University Hospital Basel, Basel, Switzerland

6: Octreopharm Science GmbH, Ipsen Group, Berlin, Germany

Supplementary Table 1: Calculated lambda values used to create the TACs by using the optimal fit function parameters from NUKFIT [1]for each species' organs and blood including standard error (SD) and coefficient of variation (CV) values determined assuming Gaussian error propagation:

Blood:

Mice Blood

	Value	SD	CV	NUKFIT function used
A1	0.4158	0.0596	0.1432	$\mathrm{A1} \cdot \mathrm{e}^{-(\lambda_1+\lambda_{\mathrm{phys}})\cdot t} + \mathrm{A2} \cdot \mathrm{e}^{-(\lambda_{\mathrm{phys}})\cdot t}$
λ_1	0.3794	0.0509	0.1342	
A2	0.0107	7.6316e-04	0.0710	

Pig Blood

	Value	SD	CV	NUKFIT function used
A1	0.0208	8.203E-04	0.0395	$\mathrm{A1} \cdot \mathrm{e}^{-(\lambda_1+\lambda_{\mathrm{phys}})\cdot t} + \mathrm{A2} \cdot \mathrm{e}^{-(\lambda_2+\lambda_{\mathrm{phys}})\cdot t} + \mathrm{A3} \cdot \mathrm{e}^{-(\lambda_3+\lambda_{\mathrm{phys}})\cdot t}$
λ_1	11.8943	0.5747	0.0483	
A2	0.0020	8.305E-05	0.0412	
λ_2	0.4066	0.0195	0.0480	
A3	1.691E-04	8.635E-06	0.0511	
λ_3	0.0076	3.076E-04	0.0405	

Human Blood

	Value	SD	CV	NUKFIT function used
A1	0.0022	4.105E-04	0.1848	$\mathrm{A1} \cdot \mathrm{e}^{-(\lambda_1+\lambda_{\mathrm{phys}})\cdot t} + \mathrm{A2} \cdot \mathrm{e}^{-(\lambda_2+\lambda_{\mathrm{phys}})\cdot t}$
λ_1	1.4713	0.3513	0.2388	
A2	6.169E-04	6.576E-05	0.1066	
λ_2	0.0609	0.0072	0.1181	

For ^{177}Lu: $\lambda_{phys} = \frac{ln(2)}{T_{1/2}} = \frac{0.6931}{159.93\ h} = 0.00435\ h$ [2].

Liver:

Mice Liver

	Value	SD	CV	NUKFIT function used
A1	1.0394	0.2313	0.2225	$A1 \cdot e^{-(\lambda_1+\lambda_{phys})\cdot t} + A2 \cdot e^{-(\lambda_{phys})\cdot t}$
λ_1	0.4724	0.1160	0.2455	
A2	0.3168	0.0183	0.0577	

Pig Liver

	Value	SD	CV	NUKFIT function used
A1	2.9475	0.4470	0.1517	$A1 \cdot e^{-(\lambda_1+\lambda_{phys})\cdot t} + A2 \cdot e^{-(\lambda_2+\lambda_{phys})\cdot t}$
λ_1	0.138	0.0669	0.4839	
A2	3.7373	0.1986	0.0531	
λ_2	0.0053	3.29E-04	0.0620	

Human Liver

	Value	SD	CV	NUKFIT function used
A1	5.4340	0.3613	0.0665	$A1 \cdot e^{-(\lambda_1+\lambda_{phys})\cdot t}$
λ_1	0.0059	0.0018	0.3005	

Kidney:

Mice Kidneys

	Value	SD	CV	NUKFIT function used
A1	2.3300	0.1580	0.0678	$A1 \cdot e^{-(\lambda_1+\lambda_{phys})\cdot t} + A2 \cdot e^{-(\lambda_{phys})\cdot t}$
λ_1	0.0183	0.0031	0.1683	
A2	0.1790	0.0679	0.3794	

Pig Kidneys

	Value	SD	CV	NUKFIT function used
A1	6.0940	0.1999	0.0328	$A1 \cdot e^{-(\lambda_1+\lambda_{phys})\cdot t} - A1 \cdot e^{-(\lambda_2+\lambda_{phys})\cdot t}$
λ_1	0.0036	2.43E-04	0.0682	
λ_2	1.7558	0.1996	0.1137	

Human Kidneys

	Value	SD	CV	NUKFIT function used
A1	4.7877	0.3183	0.0665	$A1 \cdot e^{-(\lambda_1+\lambda_{phys})\cdot t}$
λ_1	0.0038	0.0018	0.4588	

Supplementary Table 2a: The median and mean percentage injected radioactivity (%A) values per ml in blood of mice and humans with corresponding standard deviation (SD)

Time(h)	Median % A/ml Blood Mice	Mean %A /ml Blood Mice	SD
1	0.296	0.1305	0.030
4	0.099	0.0332	0.013
24	0.025	0.0085	0.005
72	0.007	0.0036	0.003
Time(h)	Median % A/ml Blood Humans	Mean %A /ml Blood Humans	SD
0.333	0.0020	0.0028	0.003
0.5	0.0016	0.0022	0.002
1	0.0010	0.0016	0.002
2	0.0007	0.0011	0.001
3	0.0006	0.0009	0.001
5	0.0004	0.0007	0.001
22.5	0.0001	0.0002	0.000

Supplementary Table 2b: The mean percentage injected radioactivity (%A) values per ml in kidneys and liver of mice and humans with corresponding standard deviation (SD)

Time(h)	Mean A% Kidneys Mice	SD
1	2.600	0.162
4	2.100	0.135
24	1.570	0.127
72	0.590	0.042
168	0.140	0.131

Time(h)	Mean A% Kidneys Humans	SD
1	5.585	2.140
3	5.303	1.713
24	4.128	1.427
72	2.678	1.072

Time(h)	Mean A% Liver Mice	SD
1	0.970	0.308
4	0.470	0.132
24	0.236	0.052
72	0.170	0.042
168	0.196	0.042

Time(h)	Mean A% Liver Humans	SD
1	4.978	2.548
3	4.480	2.289
24	4.015	1.825
72	2.688	1.148

References

[1] Kletting P, Schimmel S, Kestler HA, Hanscheid H, Luster M, Fernandez M, et al. Molecular radiotherapy: the NUKFIT software for calculating the time-integrated activity coefficient. Med Phys 2013;40:102504.
[2] M.M.Be VC, C.Dulieu, E.Browne, V.Chechev, N.Kuzmenko, R.Helmer, A.Nichols, E.Schonfeld, R.Dersch. Monographie BIPM-5 - Table of Radionuclides,. Bureau International des Poids et Mesures 2004;2:151-242.
